# Supplementary material for: Targeting PELP1 Attenuates Angiogenesis and Enhances Chemotherapy Efficiency in Colorectal Cancer
Source: Cancers (Basel). 2022 Jan 13;14(2):383. doi: 10.3390/cancers14020383 (PMC8773490; doi:10.3390/cancers14020383)
Supplement: Supplementary file 1 [file cancers-14-00383-s001.zip › cancers-1518636-supplementary.pdf]

Supplementary Material

Supplementary materials and methods

Gene set enrichment analyses

To determine the function of PELP1 in CRC, the dataset GSE29623 and GSE65979 were obtained from the Gene Expression Omnibus and analyzed by GSEA software(http://www.software. broadinstitute.org/gsea/index.jsp).The gene sets with normalized using an enrichment score of >1, p<0.05, which were regarded as significantly enriched gene sets.

Cell lines and Cell Culture

The CRC cells including HCT116,HT29,RKO,SW620 and COLO205, human immortalized normal colon epithelium (FHC) and HUVECs were obtained from the tumor cell bank of the Chinese Academy of Medical Science. All CRC cells and FHC cells were cultured in DMEM supplementedwith 10% FBS (Cat. No.10099-141, Gibco). HUVECs were cultured in basal ECM(Cat. No.1001, Sciencell) supplemented with 5% FBS and endothelial cell growth supplements. The cells were maintained at 37 °C in a humidified atmosphere of 5% CO2.

siRNA transfection

Human VEGFA siRNA and its control siRNA were purchased from Ribobio(Cat. No.CRH5078, Cohesion). The CRC cells were transfected with VEGFA siRNA and control siRNA using Lipofectamine 3000 (Cat. No.L3000015, Invitrogen) according to the manufacturer's instructions.The transfection efficiency was confirmed by the *RT-qPCR.*

Generation of stable Cell Lines

HCT116 and HT29 cells were transfected with either PELP1 overexpression plasmid (kindly provided by Dr. Ratna Vadlamudi, the University of Texas Health Science Center at San Antonio, San Antonio) and shPELP1 plasmid (Cat. No.P24031, MiaoLingBio) using Lipofectamine 3000.Stable transfectants were selected with Geneticin (Cat. No.A1720, Sigma Aldrich) or Puromycin (Cat. No.ST551, Beyotime) for 2 weeks. Resistant colonies were pooled and subcultured in the selection medium.

Immunoblot analysis

The cultured cells or xenograft tissues were harvested and lysed by RIPA buffercontaining protease inhibitors and phosphatase inhibitors.Total protein was separated by SDS-PAGE and transferred onto PVDF membranes.The membranes were blocked in 5% nonfat milk at room temperature for 1h, followed by incubation with a primary antibody against PELP1 (Cat. No.IHC-00013, Bethyl), VEGFA (Cat. No.66828-1-Ig, Proteintech), phospho-STAT3 (Cat. No.9145, CST), STAT3 (Cat. No. 4904, CST), GAPDH (Cat. No.2118S, CST) in 5% nonfat milk at 4°C overnight. After washing with TBST, the membranes were probed with secondary antibodies for 1h at room temperature. The immunoblotsignals were exposed to X-ray film (Eastman Kodak).

RT-qPCR

Total RNA was isolated from the CRC cells by TRIzol (Cat. No.Trizol, Invitrogen). 2000 ng RNA was reverse transcribed by High Capacity cDNA Reverse Transcription Kit(Cat.

No.4368814,Thermo Fisher). Then equal amount of cDNA was amplified using SYBR Green PCR amplification kit (Cat. No.4913850001,Sigma-Aldrich) with the CFX Connect Real-Time PCR Detection System (Bio-Rad, USA).GAPDH was used as an internal control. The results were normalized to GAPDH expression. Primers used are listed below.

	Forward	Reverse
PELP1	*TTGGCTTCGGAGCATTCAG*	*CCCGAGGGAAATAGGTCATAC*
VEGFA	*GTATTCAGCCAAACGACCATC*	*CTGGTTCGCTTTCTCTTTCG*
VEGFB	*GAGATGTCCCTGGAAGAACACA*	*GAGTGGGATGGGTGATGTCAG*
VEGFC	*ATGTGTGTCCGTCTACAGATGT*	*GGAAGTGTGATTGGCAAAACTGA*
VEGFD	*TCCCATCGGTCCACTAGGTTT*	*AGGGCTGCACTGAGTTCTTTG*
PGF	*GAACGGCTCGTCAGAGGTG*	*ACAGTGCAGATTCTCATCGCC*
PDGF-BB	*CTCGATCCGCTCCTTTGATGA*	*CGTTGGTGCGGTCTATGAG*
MMP2	*TACAGGATCATTGGCTACACACC*	*GGTCACATCGCTCCAGACT*
CXCL9	*CCAGTAGTGAGAAAGGGTCGC*	*AGGGCTTGGGGCAAATTGTT*
THBS1	*AGACTCCGCATCGCAAAGG*	*TCACCACGTTGTTGTCAAGGG*
PLGF	*TTTTGCCAAGGAGTGCTAAAGA*	*AACCCTCTGCACCCAGTTTTC*
Ang2	*GCTGCTGGTTTATTACTGAAGAA*	*TCAGGTGGACTGGGATGTTTAG*
EGF	*CTTGGGAGCCTGAGCAGAAA*	*TGCACAAGTGTGACTGGAGG*

Histomorphometry staining

To evaluate the hypoxia of tumor tissue, 60mg/kg PIMO (Cat. No.HP-500mgHypoxyprobe) was injected into tumor-bearing mice throughthe tail vein 90 minutes before tumor harvest, then the sections of tumor were immunostained with

anti-PIMO antibody (Cat. No.HP11-100kit, Hypoxyprobe) according to the Manufacturer's instructions. All images were captured using Bio-Tek/Cytation5 system and analyzed by Image J software (version 1.46; National Institutes of Health).

ELISA assay

The concentration of VEGFA and PDGF-BB in each CM were quantified by human VEGF ELISA kit (Cat. No.RK00023, Abclonal) and PDGF-BB ELISA kit(Cat. No. RK04112, Abclonal)accordingto the manufacturer'sprotocol.

*Cell counti*ng kit-8 assay

Cell proliferation was assessed by Cell Counting Kit-8 (CCK-8) (Cat. No.C0039, Beyotime). The transfected cells were seeded in 96-well plates at a density of 1×105 cells/well, and then added 10 μl of CCK-8 into the medium. Each plates were measured at 450 nm with a microplate reader (Bio-Tek/Cytation5 system) every 24 hours.

Animal experiment

5-week-old female nude mice were purchased from Beijing Vital River Laboratory. HCT116-shCtrl, HCT116-shPELP1 cells (5×106) were suspended at a 1:1 ratio in 200 μl PBS were injected subcutaneously into right flank of nude mice in different group respectively.To investigate whether PELP1 knockdown enhance the efficacy of chemotherapy by improve tumor vascular function in CRC, from 10th day on, we intraperitoneally injected 2.5 mg/kg of cisplatin (Cat. No. A8321, Apexbio) dissolved in PBS or PBS into the mice of different groups every other day. On the last day of treatment, nude mice were injected with 10.0 mg/kg

cisplatin and the tumor growth was monitored for 30 days. The tumor size was measured every 5 days using a slide caliper and tumor volume was calculated by the following formula: volume = 0.5× length×width2.At the end of the experiment, the mice were sacrificed and the tumors were dissected and weighed. Then the tumors were split for high performance liquid chromatography (HPLC), paraffin block, and protein extraction. All animal experiments were done according to an Institution Animal Care and Use Committee-approved protocol.

Measure the concentration of cisplatin in tumor tissue

Excess blood from the tumor tissue was wiped off, and cisplatin was separated from the tissue. The concentaration was measured by HPLC (Agilent® Technologies, Santa Clara, CA, USA) on a COSMOSIL C18 column (250 mm × 4.5 mm, 5 μm) (Shimadzu, Tokyo, Japan). Agilent ChemStation software was used in this experiment.

Statistical analyses

GraphPad Prism 5.0 (La Jolla, CA, USA) was used to analyze the all statistical data we have obtained. Student's t-test was used to find the differences between groups. The correlation between PELP1 with CD31, CD34 and VEGFA expression of CRC patients was analyzed by Pearson's correlation test. All experiments were performed in triplicate, and $p < 0.05$ was considered statistically significant.

Supplementary figures and legends

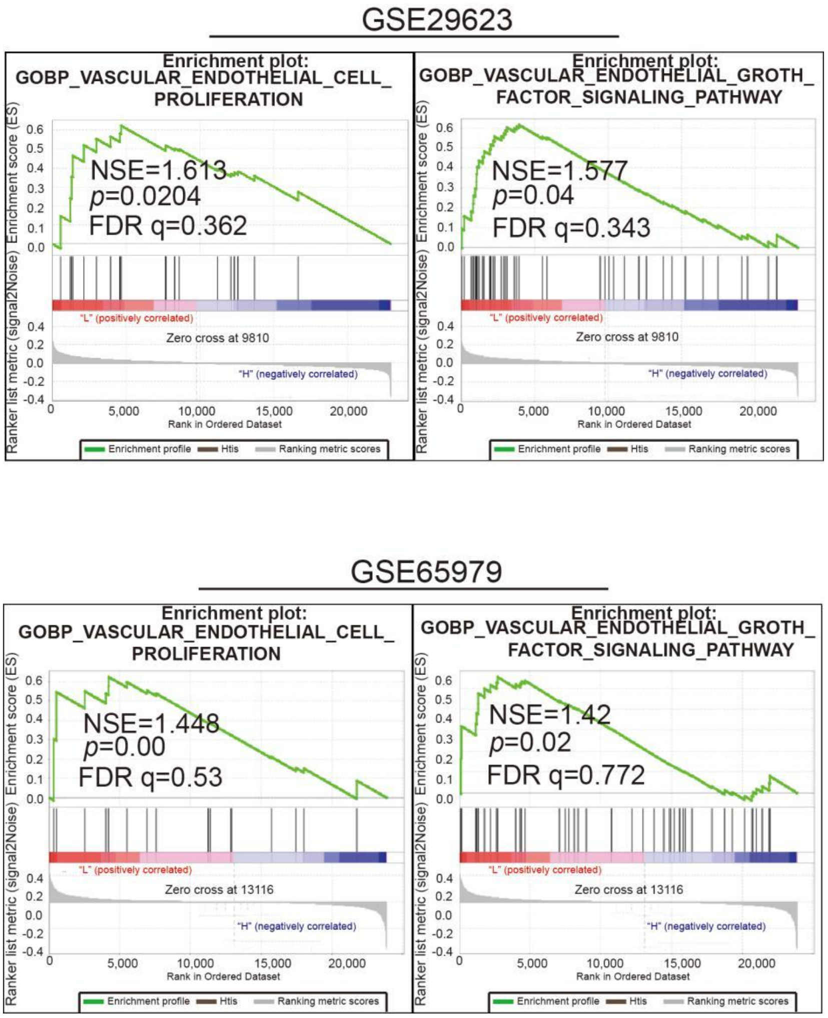

Figure S1. PELP1 is positively correlated with angiogenesis in CRC. GSEA of enrichment of GOBP_VASCULAR_ENDOTHELIAL_CELL_PROLIFERATIONand GOBP_VASCULAR_ENDOTHELIAL_GROWTH_FACTOR_SIGNALING_PATHWAY in high expression versus low expression of PELP1 in GSEGSE29623 and GSE65979 dataset. FDR, false-discovery rate q value. NES, normalized enrichment score.

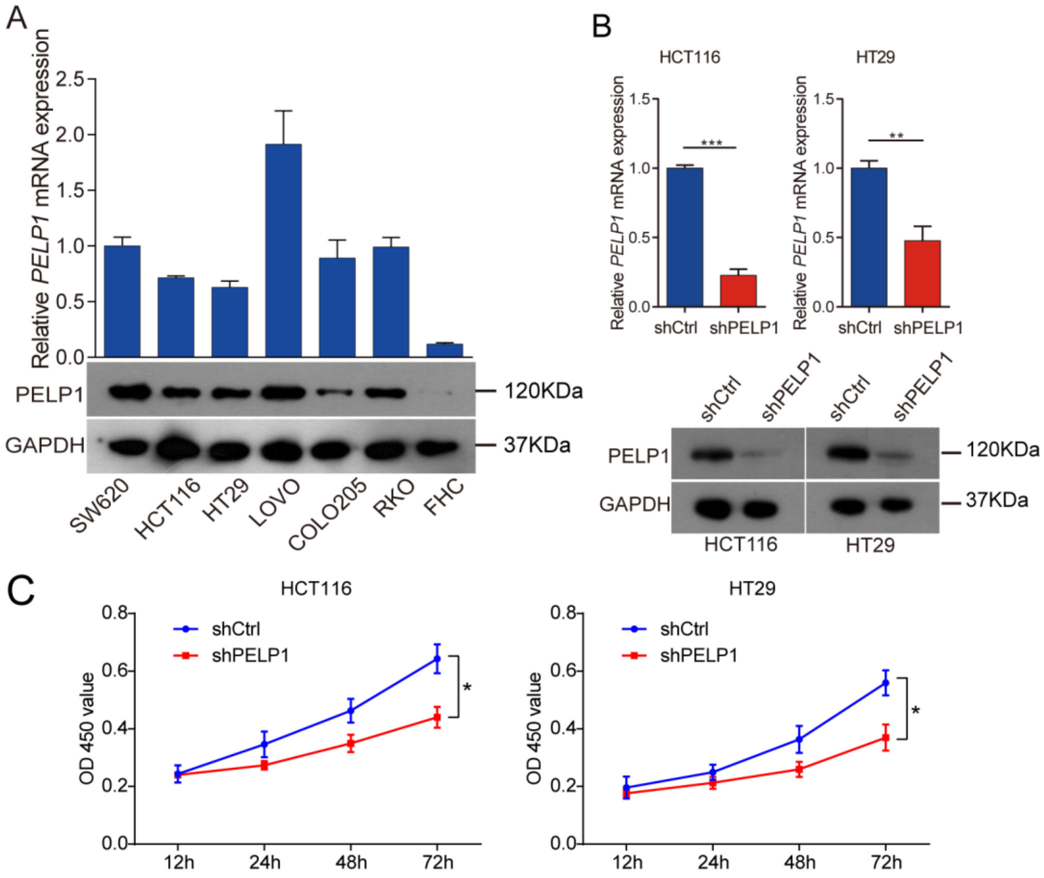

Figure S2. PELP1 is highly expressed in CRC cells. (**A**) RT-qPCR (upper) and immunoblot (lower) analysis of PELP1 in CRC cell lines and immortalized colonic epithelium (FHC). GAPDH was used as an internal control. (**B**) RT-qPCR (upper) and immunoblot (lower) of PELP1 expression in HCT116 and HT29 cells transfected with either control shRNA (shCtrl) or shRNA against PELP1 (shPELP1). GAPDH was used as an internal control. (**C**) CCK8 assays for HCT116 and HT29 cells transfected with either control shRNA or shRNA against PELP1. Data are shown as the means of three independent experiments or representative data. Error bars indicate SD.*$p < 0.05$, **$p < 0.01$, ***$p < 0.001$ by Student's t-test.

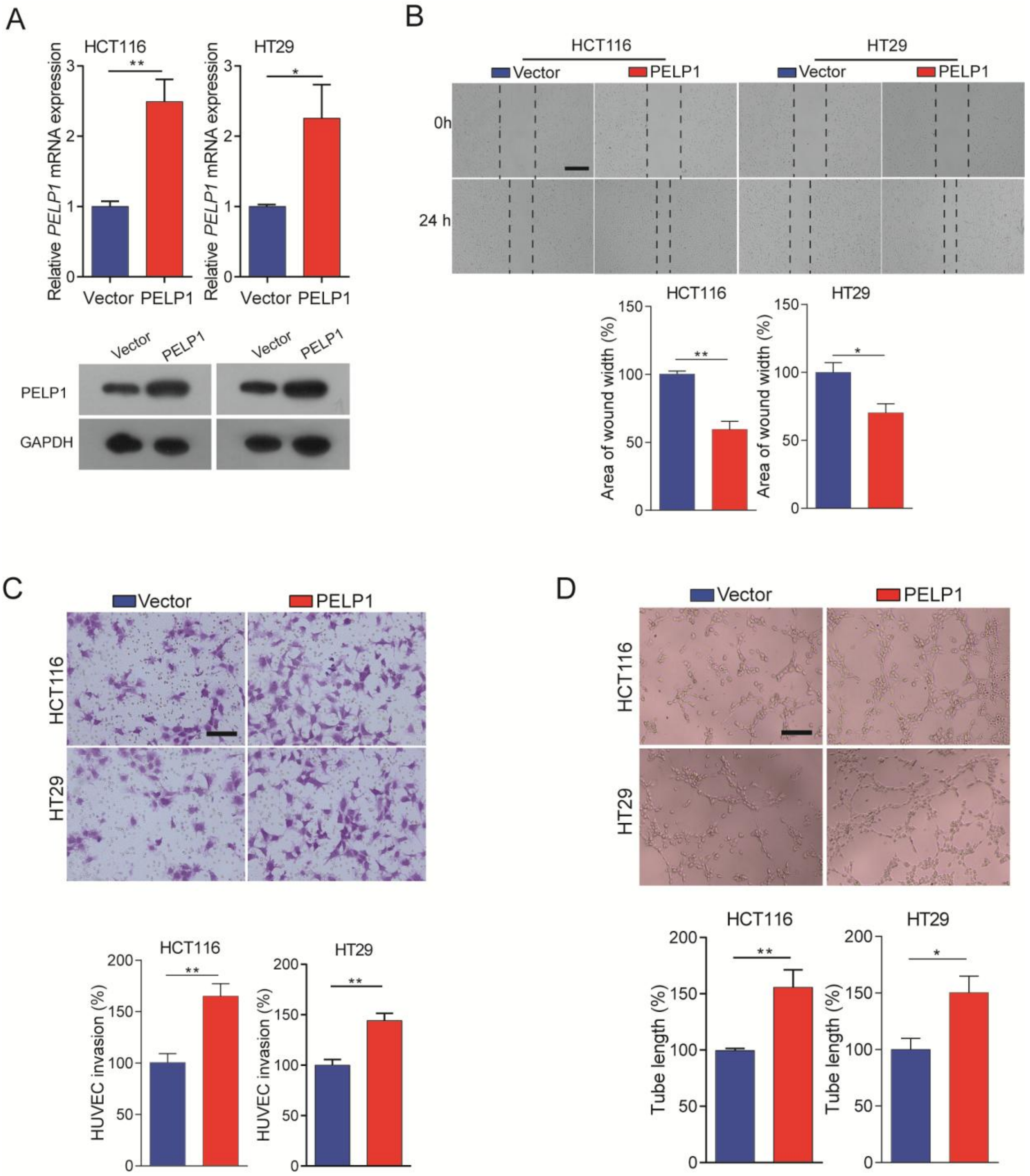

Figure S3. Overexpression of PELP1 enhances angiogenesis in vitro. (**A**) RT-qPCR (upper panel) and immunoblot (lower panel) analysis of PELP1 in PELP1 overexpression cells. GAPDH was used as an internal control. (**B**) Representative images of wound healing in HUVECs treated with CM from PELP1 CRC cells (upper panel). Scale bar: 200 μm. Histograms with the fold change in wound closure formed by the indicated cells (lower panel). (**C**) Representative images of cell invasion in HUVECs treated with CM from PELP1 CRC cells (upper panel). Scale bar: 200 μm. Histograms with the fold change in the number of

invaded cells formed by the indicated cells (lower panel). (**D**) Representative images of tube formation in HUVECs treated with CM from PELP1 CRC cells (upper panel). Scale bar: 200 μm. Histograms with the fold change in the length of tube-like formation formed by the indicated cells (lower panel). Data are shown as the means of three independent experiments or representative data. Error bars indicate SD.*p < 0.05,**p < 0.01, ***p < 0.001 by Student's t-test.

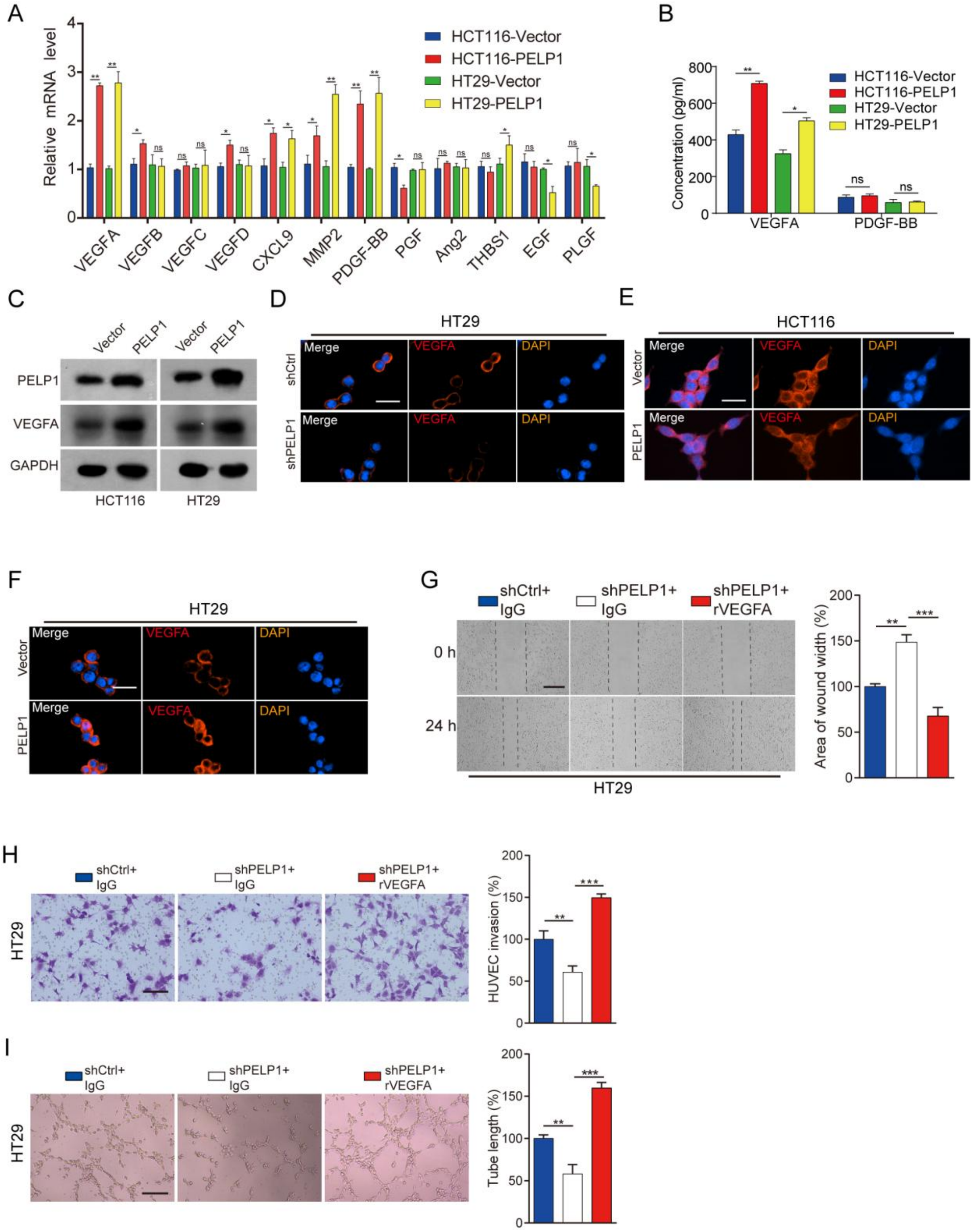

Figure S4. VEGFA is a critical key for PELP1 to promote angiogenesis in CRC. (**A**) The mRNA levels of the 12 angiogenesis-related genes in PELP1-overexpressing cells. GAPDH was used as an internal control. (**B**) ELISA analysis of VEGFA and PDGF-BB expression in PELP1-overexpressing CRC cells. (**C**) Immunoblot analysis of PELP1 and VEGFA expressions in PELP1-overexpressing CRC cells. GAPDH was used as an internal control. (**D**)

Representative images of immunofluorescence for VEGFA in HT29-shCtrlCRC cells or HT29-shPELP1CRC cells. Scale bar: 30 μm. (**E, F**) Representative images of immunofluorescence for VEGFA in PELP1-overexpressing CRC cells. Scale bar: 30 μm. (**G**) Representative images of wound healing in HUVECs treated with CM from HT29-shCtrl CRC cells or HT29-shPELP1 CRC cells added with or without recombinant VEGFA (rVEGFA) and IgG (left panel). Scale bar: 200 μm. Histograms with the fold change in wound closure formed by the indicated cells (right panel). (**H**) Representative images of cells migration in HUVECs treated with CM from HT29-shCtrl CRC cells or HT29-shPELP1 CRC cells added with or without rVEGFA and IgG (left panel). Scale bar: 200 μm. Histograms with the fold change in the number of invasion cells formed by the indicated cells (right panel). (**I**) Representative images of tube formation in HUVECs treated with CM from HT29-shCtrl CRC cells or HT29-shPELP1 CRC cells added with or without rVEGFA and IgG (left panel). Scale bar: 200 μm. Histograms with the fold change in the length of tube-like formation formed by the indicated cells (right panel). Data are shown as the means of three independent experiments or representative data.Error bars indicate SD.n.s.: no significance, $*p < 0.05$, $**p < 0.01$, $***p < 0.001$ by Student's t-test.

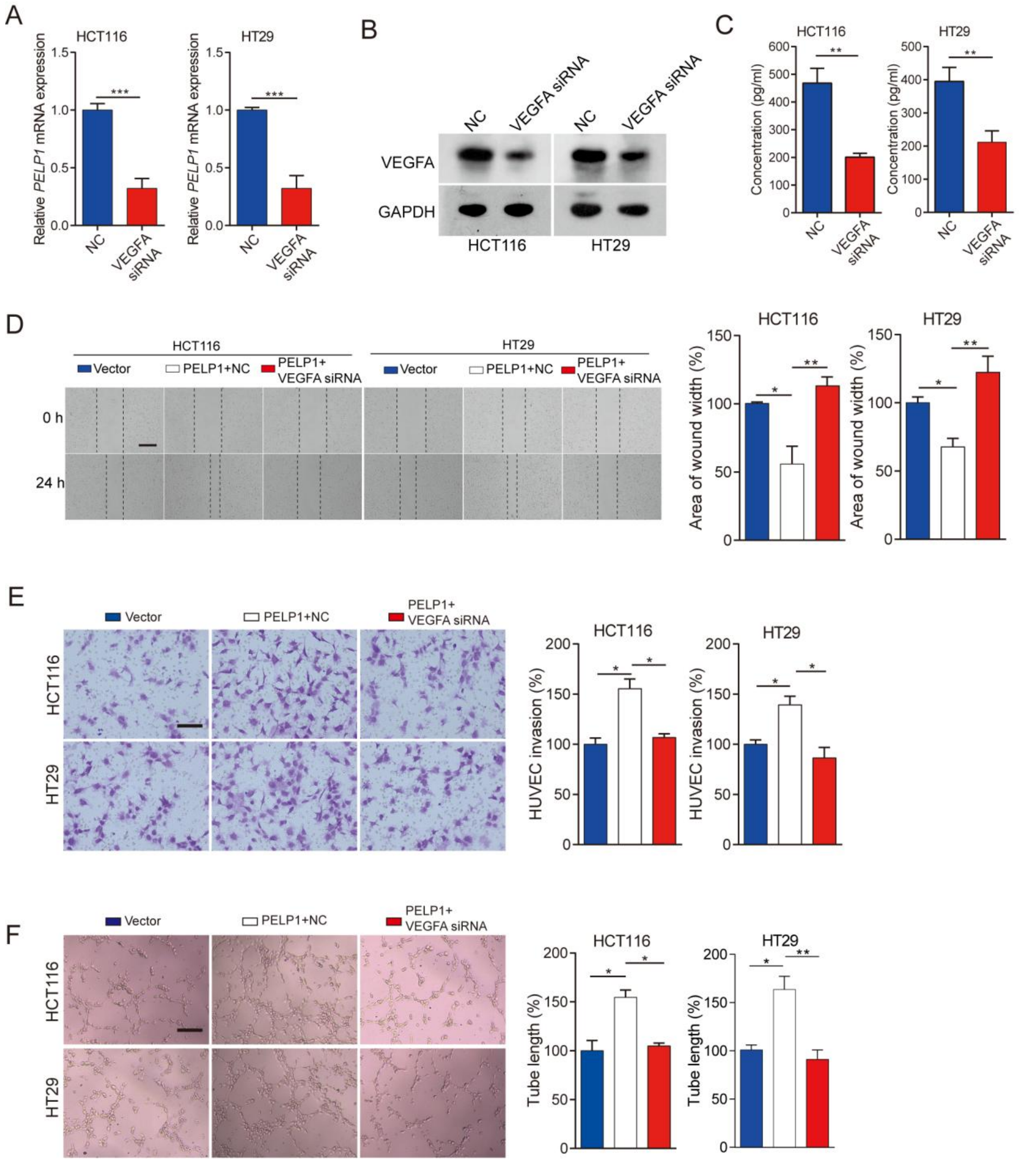

Figure S5. Downregulation of VEGFA inhibits PELP1-mediated angiogenesis. (**A-C**) RT-qPCR (A), immunoblot (B) and ELISA (C) of PELP1 expression in PELP1-overexpressing CRC cells transfected with siRNA negative control (NC) or VEGFA siRNA. (**D**) Representative images of wound healing in HUVECs treated with CM from PELP1-overexpressing CRC cells transfected with siRNA negative control (NC) or VEGFA siRNA (left panel). Scale bar: 200 μm. Histograms with the fold change in wound closure formed by the indicated cells (right panel). (**E**) Representative images of cell migration in HUVECs treated with CM from

PELP1-overexpressing CRC cells transfected with siRNA negative control (NC) or VEGFA siRNA (left panel). Scale bar: 200 μm. Histograms with the fold change in the number of migrated cells formed by the indicated cells (right panel). (**F**) Representative images of cell migration in HUVECs treated with CM from PELP1-overexpressing CRC cells transfected with siRNA negative control (NC) or VEGFA siRNA (left panel). Scale bar: 200 μm. Histograms with the fold change in the length of tube-like formation formed by the indicated cells (right panel). Data are shown as the means of three independent experiments or representative data. Error bars indicate SD. *$p < 0.05$, **$p < 0.01$, ***$p < 0.001$ by Student's t-test.

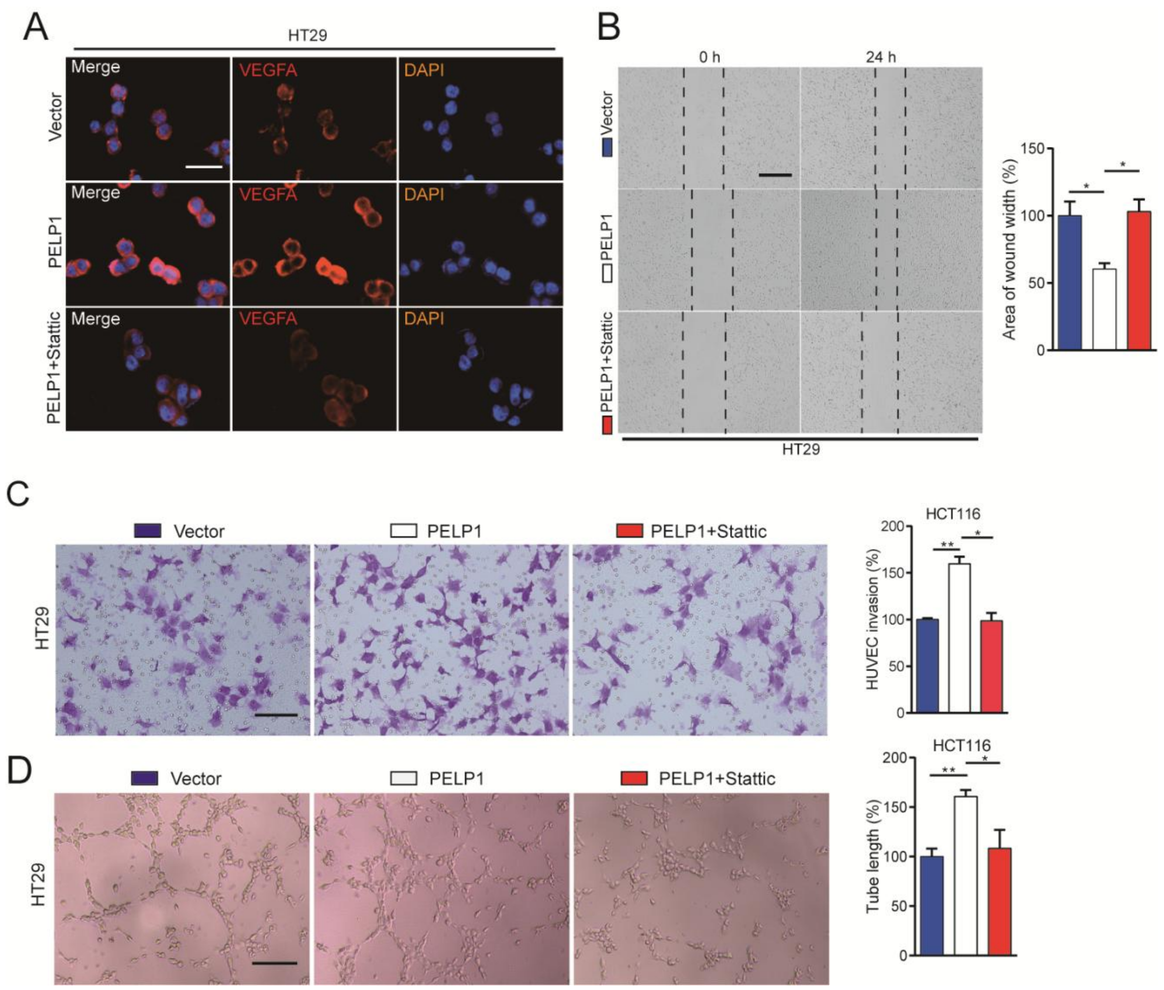

Figure S6. PELP1 promotes angiogenesis via STAT3/VEGFA axis in vitro. (**A**) Representative images of immunofluorescence for VEGFA in HT29-PELP1 CRC cells treated with or without Stattic. Scale bar: 30 μm. (**B**) Representative images of wound healing in HUVECs treated with CM from HT29-PELP1 cells treated with or without Stattic (left panel). Scale bar: 200 μm. Histograms with the fold change in wound closure formed by the indicated cells (right panel).(**C**) Representative images of cell invasion in HUVECs treated with CM from HT29-PELP1cells treated with or without Stattic (left panel). Scale bar: 200 μm. Histograms with the fold change in the number of invaded cells formed by the indicated cells (right panel). (**D**) Representative images of tube formation in HUVECs treated with CM from HT29-PELP1 CRC cells treated with or without Stattic (left panel). Scale bar: 200 μm.

Histograms with the fold change in the length of tube-like formation formed by the indicated cells (right panel). Data are shown as the means of three independent experiments or representative data. Error bars indicate SD.*$p < 0.05$,**$p < 0.01$, ***$p < 0.001$ by Student's t-test.

Densitometry readings/intensity ratio of each band of total blot

Figure. 3

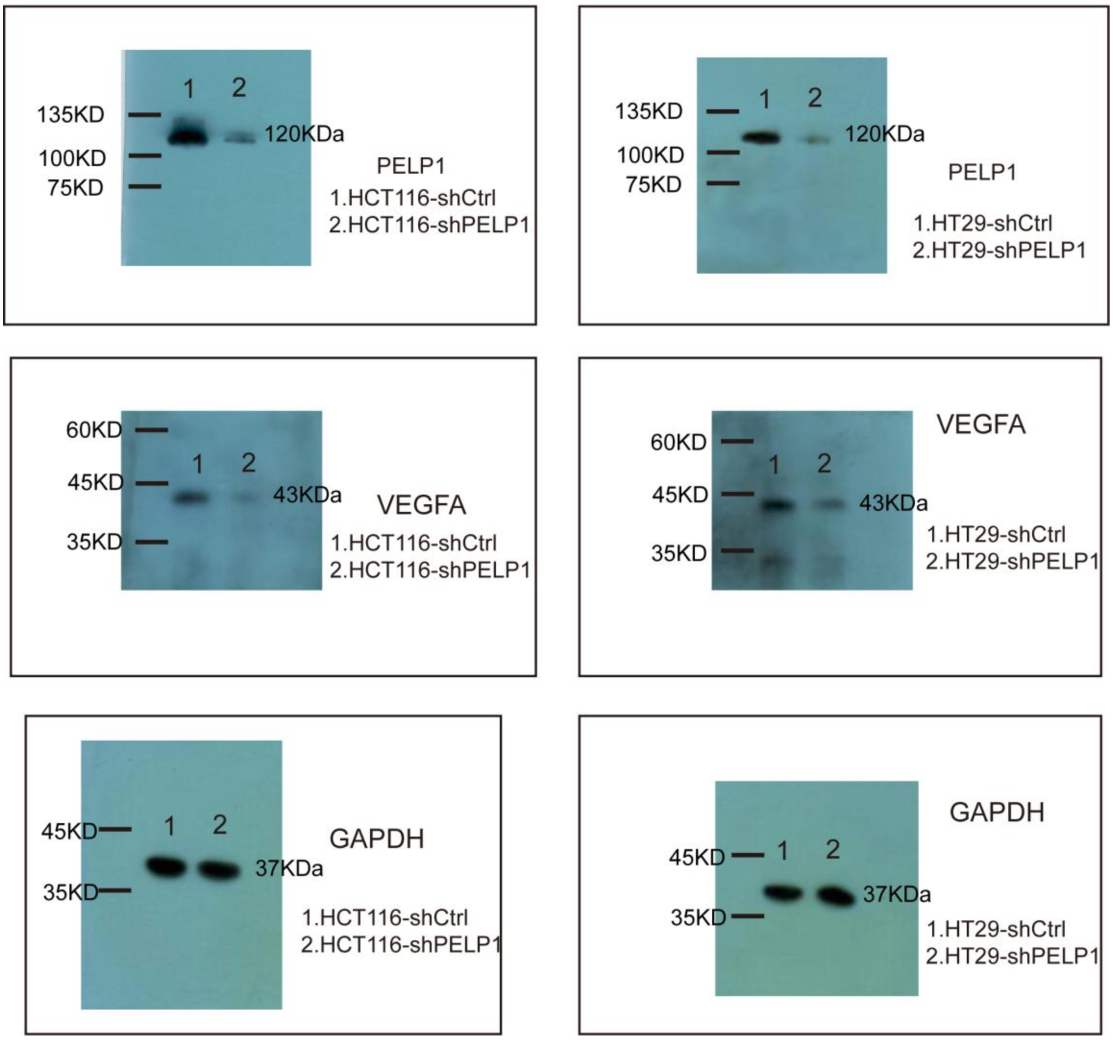

Figure S7

Figure 3 CRC cells GAPDH loading control

	**PELP1**	**GAPDH**
**HCT116-Vector**	**537325**	**601795**
**HCT116-PELP1**	**818556**	**580956**
**HT29-Vector**	**564462**	**427336**
**HT29-PELP1**	**755344**	**405240**

Ration on loading control

HCT116-Vector	HCT116-PELP1	HT29-Vector	HT29-PELP1
0.892870496	1.408981059	1.320885673	1.863942355
HCT116-Vector mean		0.892870496	
	Relative Unit on control mean		
1	1.578035	1.47937	2.087584

Figure 4A

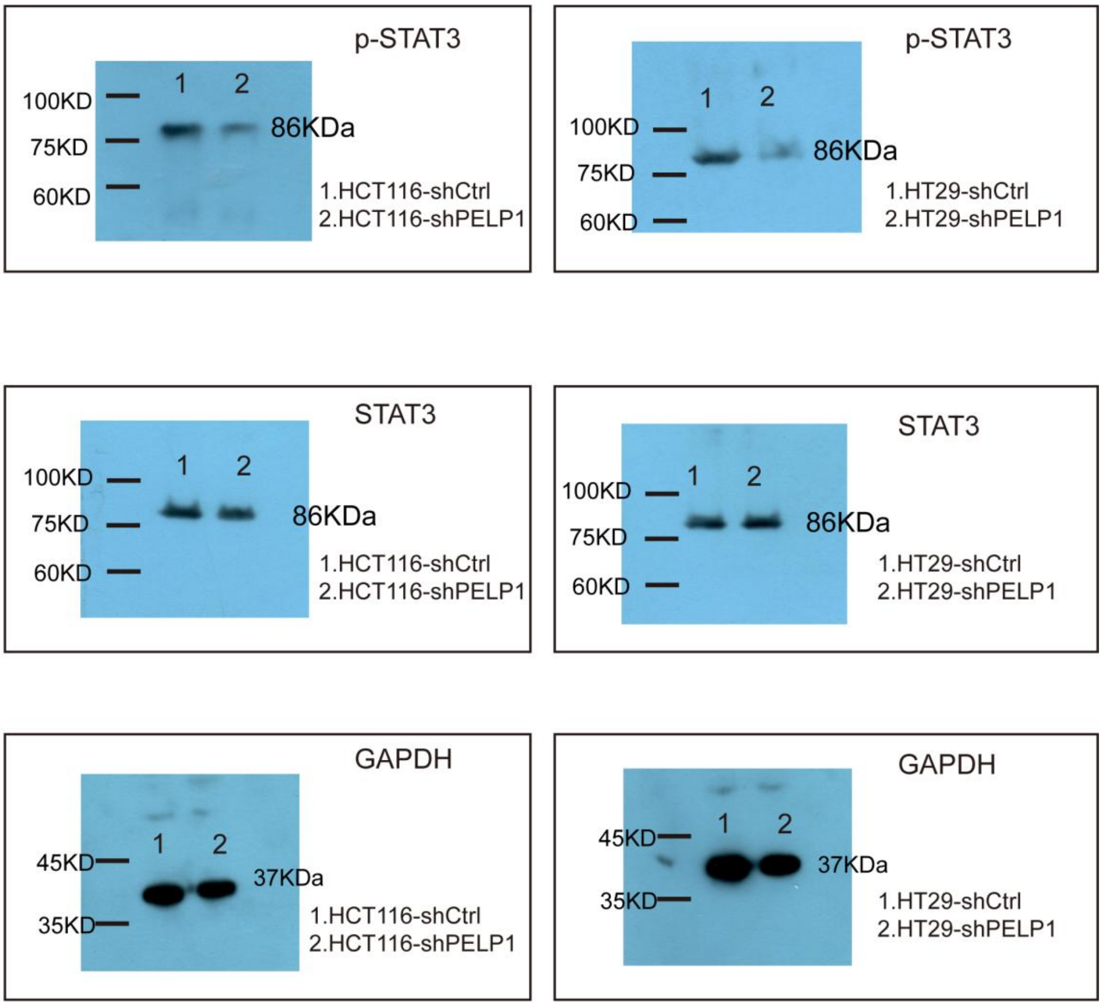

Figure S8

Figure 4A CRC cells GAPDH loading control

	**P-STAT3**	**GAPDH**	
**HCT116-shCtrl**	**585575**	**684620**	**0.855328503**
**HCT116-shPELP1**	**100477**	**719028**	**0.139740038**
**HT29-shCtrl**	**542239**	**629611**	**0.8612286**
**HT29-shPELP1**	**131942**	**651488**	**0.202524068**

Ration on loading control

HCT116-shCtrl	HCT116-shPELP1	HT29-shCtrl	HT29-shPELP1
0.855328503	0.139740038	0.8612286	0.202524068

HCT116-shCtrl mean	0.855328503

Relative Unit on control

HCT116-shCtrl	HCT116-shPELP1	HT29-shCtrl	HT29-shPELP1
1	0.163376	1.006898	0.236779

	STAT3	GAPDH	
HCT116-shCtrl	611062	684620	0.892556455
HCT116-shPELP1	609589	719028	0.847795913
HT29-shCtrl	686923	629611	1.091027635
HT29-shPELP1	673923	651488	1.034436551

Ration on loading control

HCT116-shCtrl	HCT116-shPELP1	HT29-shCtrl	HT29-shPELP1
0.892556455	0.847795913	1.091027635	1.034436551

HCT116-shCtrl mean	0.892556455

Relative Unit on control

HCT116-shCtrl	HCT116-shPELP1	HT29-shCtrl	HT29-shPELP1
1	0.949851	1.222363	1.158959

Figure 4C

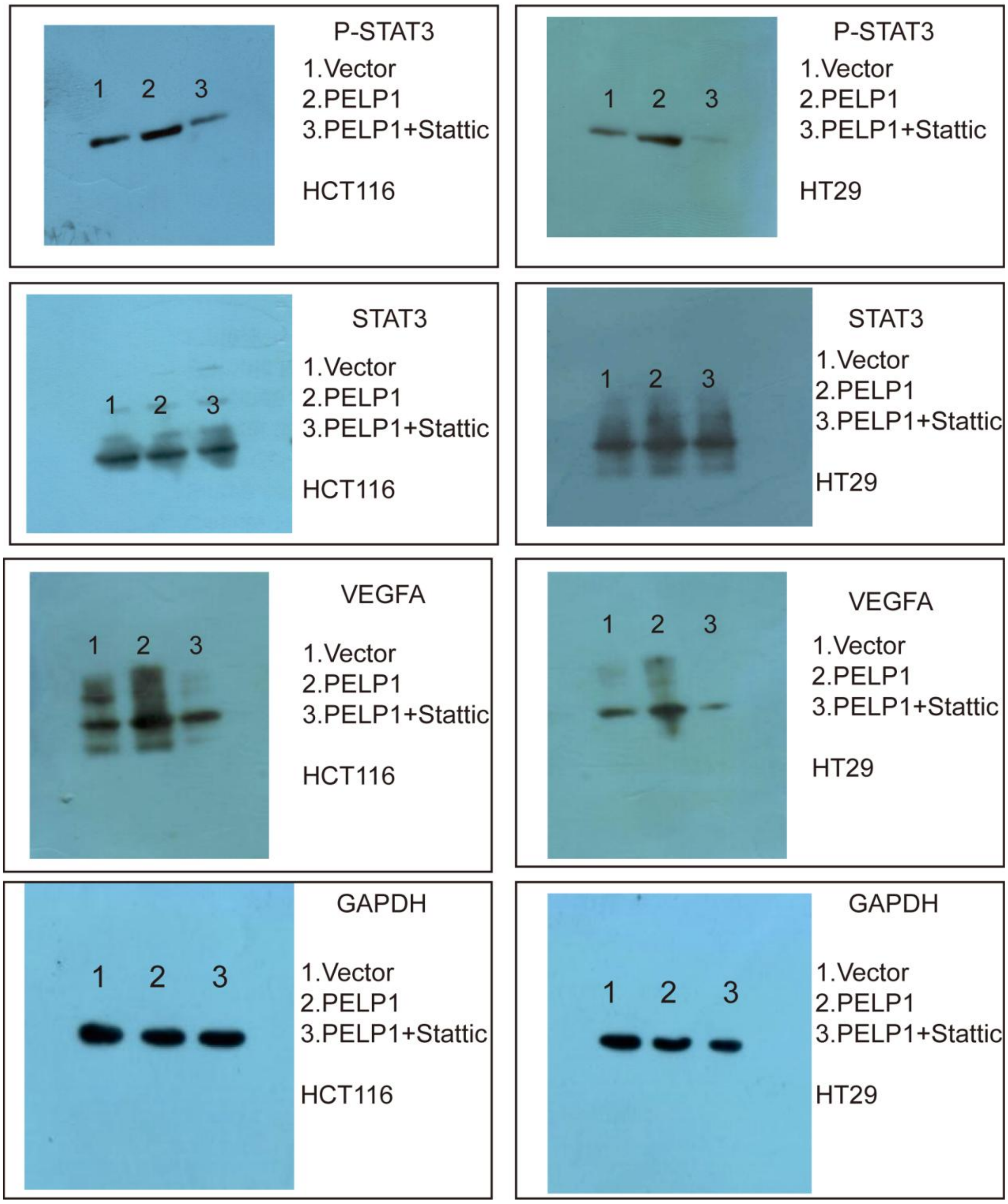

Figure S9

Figure 4C CRC cells GAPDH loading control

	p-STAT3	GAPDH
HCT116-Vector	437125	655700
HCT116-PELP1	701251	638493

HCT116-PELP1+Stattic	263473	622217
HT29-Vector	266668	478213
HT29-PELP1	464788	471733
HT29-PELP1+Stattic	121381	493048

Ration on loading control

HCT116-Vector	HCT116-PELP1	HCT116-PELP1+Stattic	HT29-Vector	HT29-PELP1	HT29-PELP1+Stattic
0.666653958	1.09829081	0.423442304	0.5576343	0.9852776	0.246184956

HCT116-shCtrl mean **0.666653958**

Relative Unit on control

HCT116-Vector	HCT116-PELP1	HCT116-PELP1+Stattic	HT29-Vector	HT29-PELP1	HT29-PELP1+Stattic
1	1.647468	0.635176	0.836467	1.477945	0.369284

	STAT3	GAPDH
HCT116-Vector	696842	655700
HCT116-PELP1	701251	638493
HCT116-PELP1+Stattic	716934	622217
HT29-Vector	475935	478213
HT29-PELP1	464788	471733
HT29-PELP1+Stattic	465592	493048

Ration on loading control

HCT116-Vector	HCT116-PELP1	HCT116-PELP1+Stattic	HT29-Vector	HT29-PELP1	HT29-PELP1+Stattic
1.062745158	1.09829081	1.152225028	0.995236	0.985277	0.944313738

HCT116-shCtrl mean	1.062745158

Relative Unit on control

HCT116-Vector	HCT116-PELP1	HCT116-PELP1+Stattic	HT29-Vector	HT29-PELP1	HT29-PELP1+Stattic
1	1.033447	1.084197	0.936477	0.927106	0.888561

	VEGFA	GAPDH
HCT116-Vector	380740	655700
HCT116-PELP1	664250	638493
HCT116-PELP1+Stattic	308280	622217

HT29-Vector	251900	478213
HT29-PELP1	554030	471733
HT29-PELP1+Stattic	188620	493048

Ration on loading control

HCT116-Vector	HCT116-PELP1	HCT116-PELP1+Stattic	HT29-Vector	HT29-PELP1	HT29-PELP1+Stattic
0.580661888	1.04034030	0.495454158	0.5267527	1.1744567	0.382559102

HCT116-shCtrl mean	0.580661888

Relative Unit on control

HCT116-Vector	HCT116-PELP1	HCT116-PELP1+Stattic	HT29-Vector	HT29-PELP1	HT29-PELP1+Stattic
1	1.791646	0.853258	0.907159	2.022617	0.658833

Figure 5

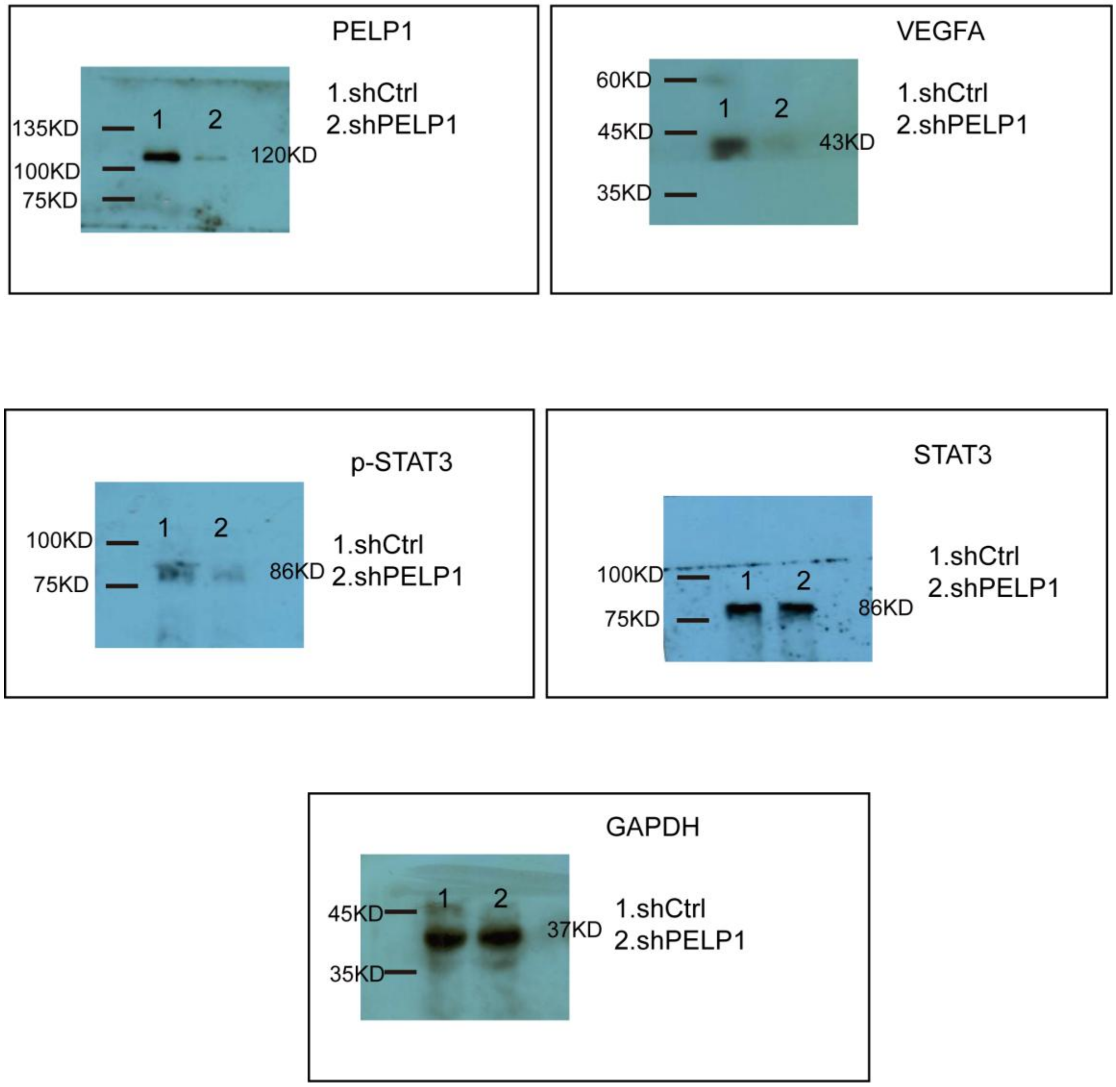

Figure S10

Figure 5 CRC cells GAPDH loading control

	VEGFA	GAPDH
HCT116-shCtrl	288635	385212
HCT116-shPELP1	47346	380897

Ration on loading control

HCT116-shCtrl	HCT116-shPELP1

0.749288703	0.12430132
HCT116-shCtrl mean	0.749288703

Relative Unit on control

HCT116-shCtrl	HCT116-shPELP1
1	0.165892

	p-STAT3	GAPDH
HCT116-shCtrl	321647	385212
HCT116-shPELP1	79959	380897

Ration on loading control

HCT116-shCtrl	HCT116-shPELP1
0.834986968	0.209922893
HCT116-shCtrl mean	0.834986968

Relative Unit on control

HCT116-shCtrl	HCT116-shPELP1
1	0.251409

	STAT3	GAPDH
HCT116-shCtrl	589015	385212
HCT116-shPELP1	605874	380897

Ration on loading control

HCT116-shCtrl	HCT116-shPELP1

**1.529067111**	**1.590650491**

**HCT116-shCtrl mean**	**1.529067111**

Relative Unit on control

**HCT116-shCtrl**	**HCT116-shPELP1**
**1**	**1.040275**

Figure S2A

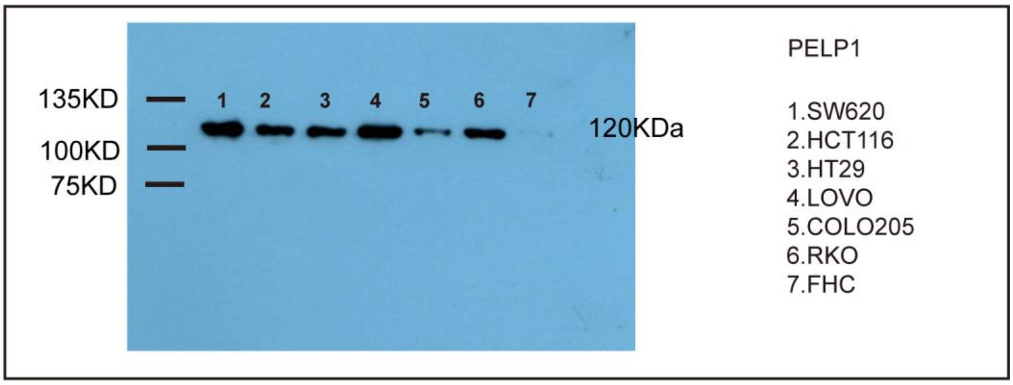

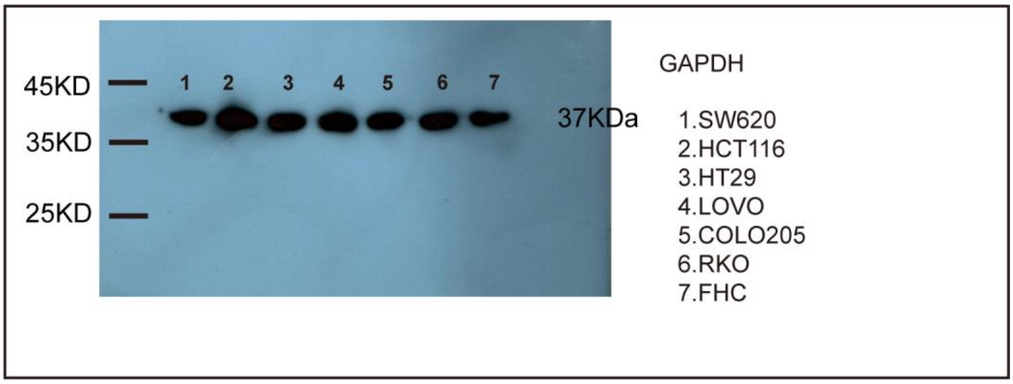

Figure S11

Figue S2A CRC cells GAPDH loading control

	PELP1	GAPDH
SW620	1065298	640401
HCT116	665102	638276
HT29	668618	592436
LOVO	1066366	626363
COLO205	224449	652720
RKO	655692	625395
FHC	7127	607894

Ration on loading control	SW620	HCT116	HT29	LOVO	COLO205	RKO	FHC
	1.663485847	1.04202884	1.128591105	1.702472847	0.343867202	1.048444583	0.011724083

SW620 mean	1.663485847

Relative Unit on control	SW620	HCT116	HT29	LOVO	COLO205	RKO	FHC
	1	0.626413	0.678449	1.023437	0.206715	0.63027	0.007048

Figure S2B

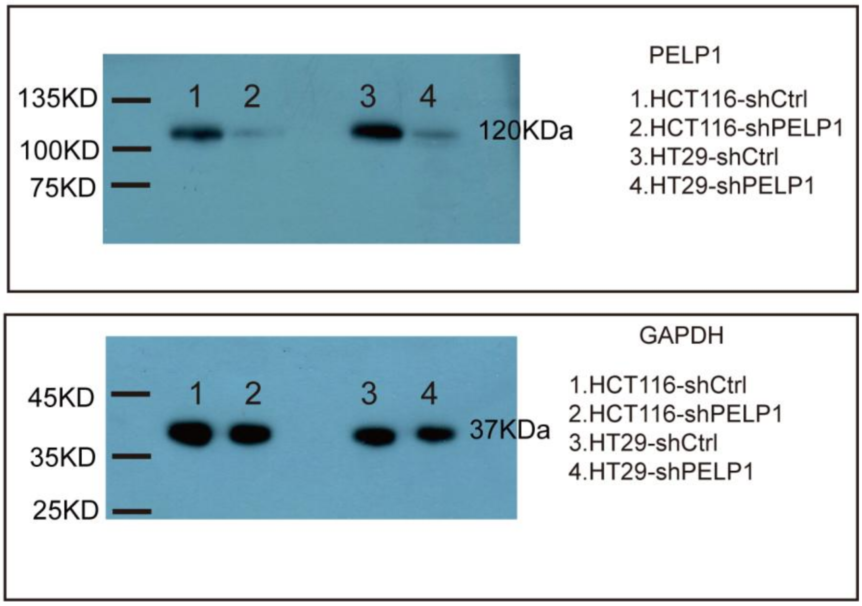

Figure S12

Fiugre S2B CRC cells GAPDH loading control

	PELP1	GAPDH
HCT116-shCtrl	593860	793883
HCT116-shPELP1	38630	824244
HT29-shCtrl	741506	832120
HT29-shPELP1	105435	755960

Ration on loading control

HCT116-shCtrl	HCT116-shPELP1	HT29-shCtrl	HT29-shPELP1
0.748044737	0.04686719	0.891104648	0.139471665

HCT116-shCtrl mean **0.748044737**

Relative Unit on control mean

HCT116-shCtrl	HCT116-shPELP1	HT29-shCtrl	HT29-shPELP1

**1**	**0.062653**	**1.191245**	**0.186448**

Fiugre S3A

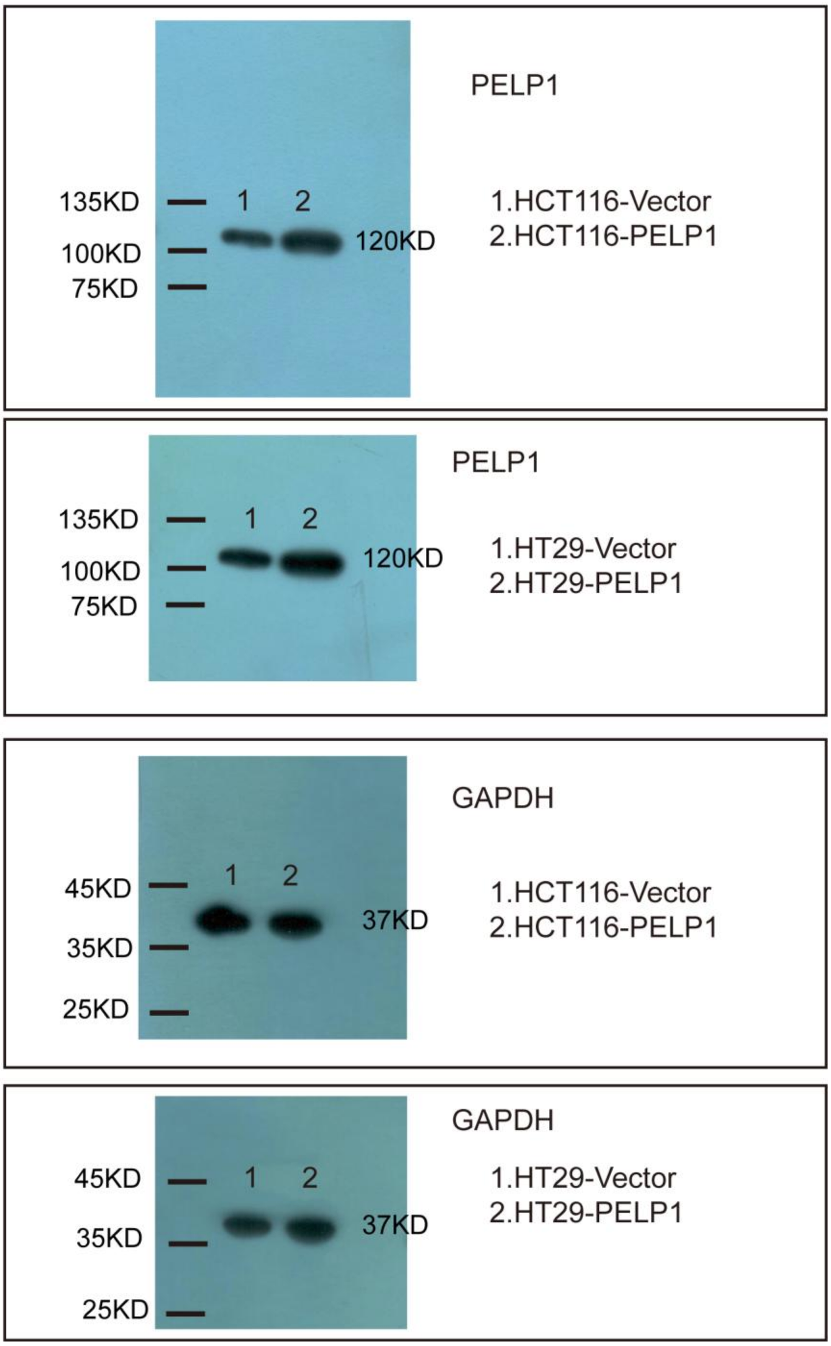

Figure S13

Figure S3A CRC cells GAPDH loading control

	PELP1	GAPDH
HCT116-Vector	537325	601795
HCT116-PELP1	818556	580956

HT29-Vector		564462	427336
HT29-PELP1		755344	405240

Ration on loading control

HCT116-Vector	HCT116-PELP1	HT29-Vector	HT29-PELP1
0.892870496	1.408981059	1.320885673	1.863942355
HCT116-Vector mean		0.892870496	
	Relative Unit on control mean		
1	1.578035	1.47937	2.087584

Fiugre S4C

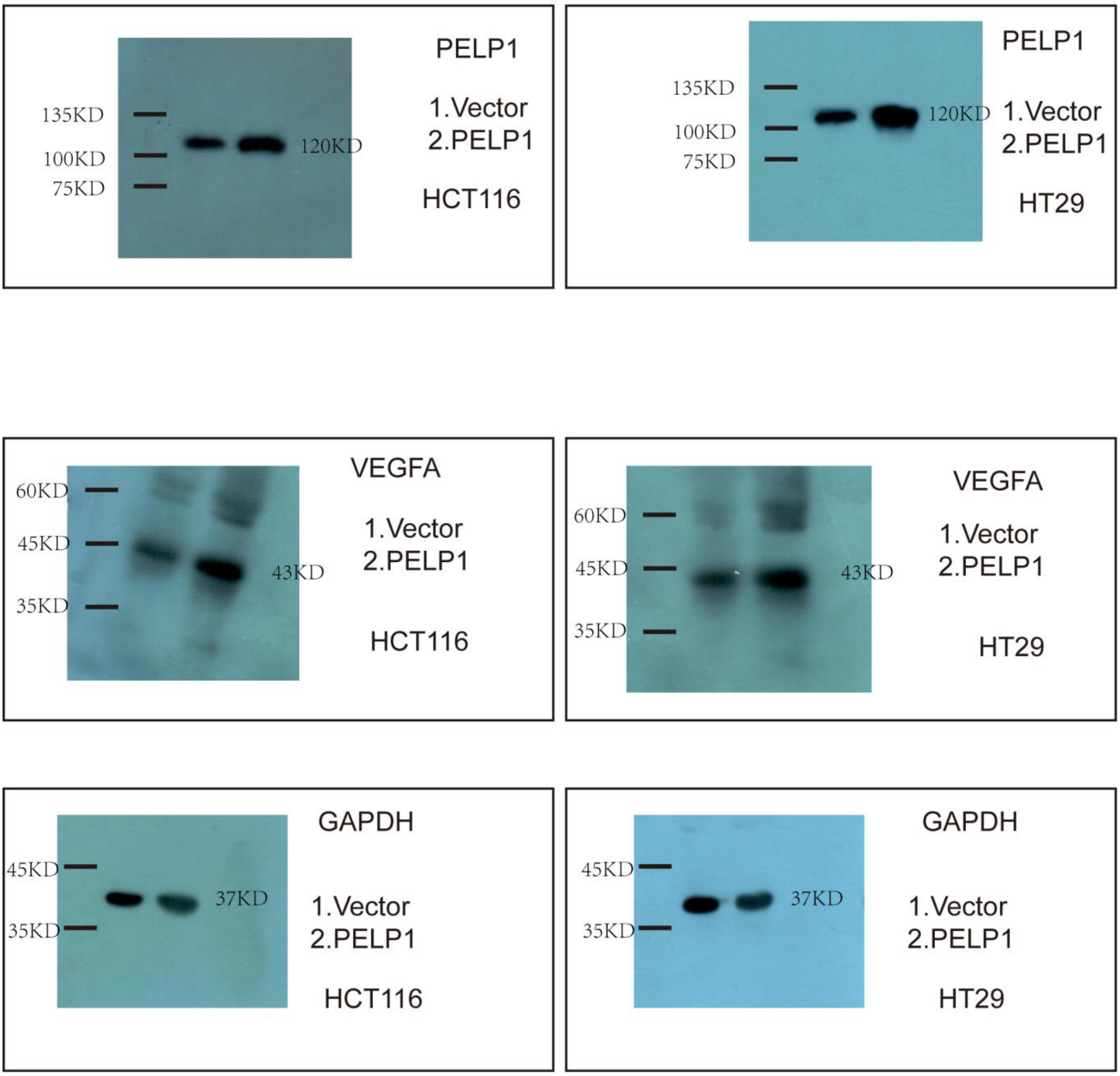

Figure S14

Figure S4C CRC cells GAPDH loading control

	PELP1	GAPDH	
**HCT116-Vector**	**359502**	**414325**	**1.045763591**
**HCT116-PELP1**	**522996**	**468304**	**0.422970976**

HT29-Vector	577792	615900	0.670214321
HT29-PELP1	724212	594358	0.315552243

Ration on loading control

HCT116-Vector	HCT116-PELP1	HT29-Vector	HT29-PELP1
1.045763591	0.422970976	0.670214321	0.315552243

HCT116-shCtrl mean 1.045763591

Relative Unit on control

HCT116-Vector	HCT116-PELP1	HT29-Vector	HT29-PELP1
1	0.404461	0.640885	0.301743

	VEGFA	GAPDH	
HCT116-Vector	157986	414325	0.381309359
HCT116-PELP1	341720	468304	0.729696949
HT29-Vector	161679	615900	0.262508524
HT29-PELP1	318165	594358	0.535308686

Ration on loading control

HCT116-Vector	HCT116-PELP1	HT29-Vector	HT29-PELP1
0.381309359	0.729696949	0.262508524	0.535308686

HCT116-shCtrl mean 0.381309359

Relative Unit on control

HCT116-Vector	HCT116-PELP1	HT29-Vector	HT29-PELP1
1	1.913661	0.68844	1.40387

Figure S5

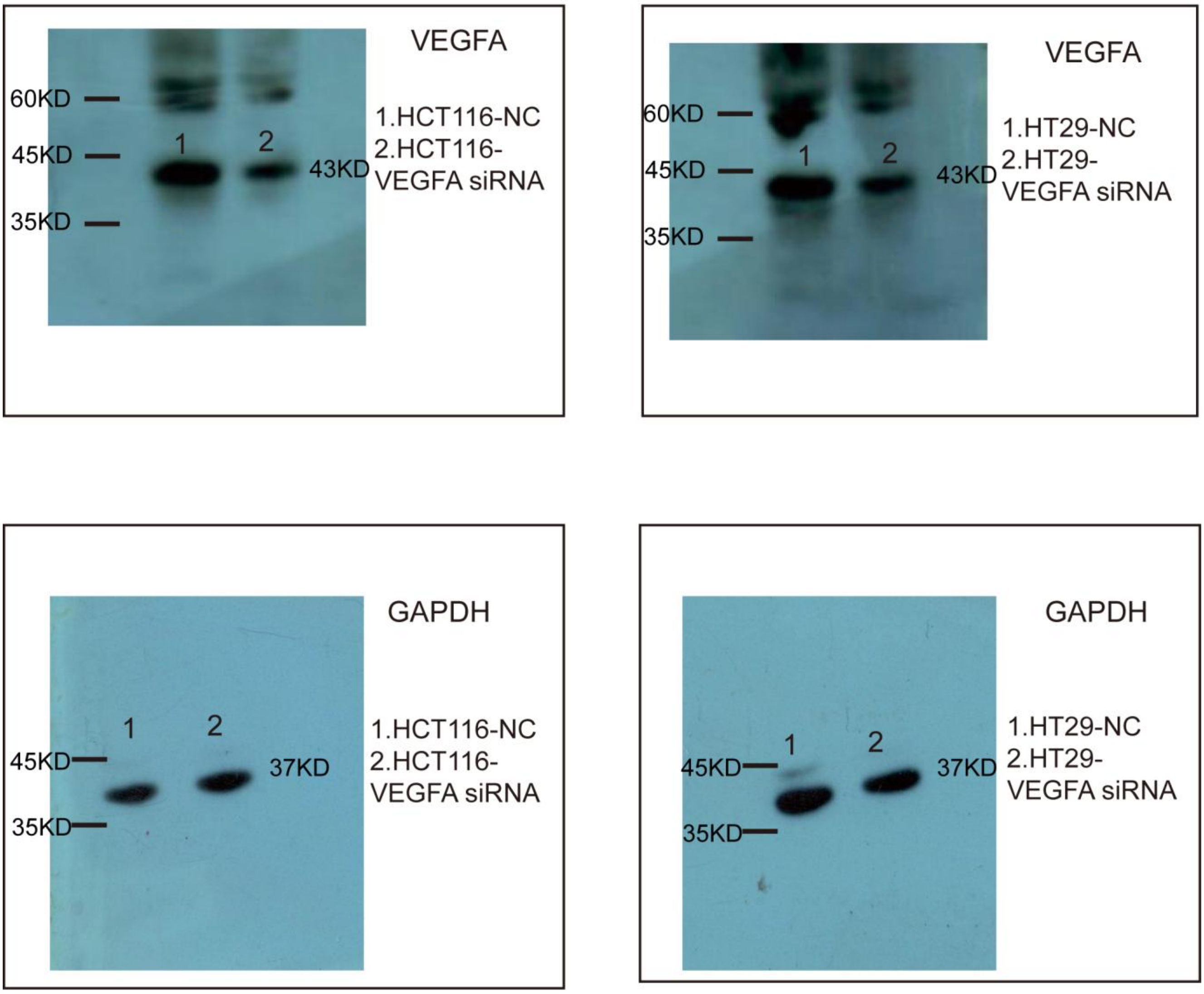

Figure S15

Figure S5 CRC cells GAPDH loading control

	VEGFA	GAPDH	
HCT116-NC	433286	414325	1.045763591
HCT116-VEGFA siRNA	198079	468304	0.422970976
HT29-NC	412785	615900	0.670214321
HT29-VEGFA siRNA	187551	594358	0.315552243

Ration on loading control

HCT116-shCtrl	HCT116-shPELP1	HT29-shCtrl	HT29-shPELP1
1.045763591	0.422970976	0.670214321	0.315552243

HCT116-shCtrl mean	1.045763591

Relative Unit on control

HCT116-shCtrl	HCT116-shPELP1	HT29-shCtrl	HT29-shPELP1
1	0.404461	0.640885	0.301743
